# Deletion of the *foxO4* Gene Increases Hypoxia Tolerance in Zebrafish

**DOI:** 10.3390/ijms24108942

**Published:** 2023-05-18

**Authors:** Linlin Shi, Axin Zhang, Hong Liu, Huanling Wang

**Affiliations:** 1 Key Lab of Freshwater Animal Breeding/Key Laboratory of Agricultural Animal Genetics, Breeding and Reproduction, Ministry of Education, College of Fishery, Huazhong Agricultural University, Wuhan 430070, China; 2 Engineering Research Center of Green Development for Conventional Aquatic Biological Industry in the Yangtze River Economic Belt, Ministry of Education, Wuhan 430070, China

**Keywords:** *foxO4*, hypoxia, zebrafish, oxygen consumption

## Abstract

Oxygen homeostasis is an important organizing principle for understanding development, physiology, disease, and evolution. Under various physiological and pathological states, organisms experience oxygen deficiency or hypoxia. FoxO4 has been recognized as an important transcriptional regulator involved in a variety of cellular functions, including proliferation, apoptosis, differentiation, and stress resistance, but its role in hypoxia adaptation mechanisms in animals is not so clear. To explore the role of *foxO4* in the hypoxia response, we detected the expression of *foxO4* and the regulatory relationship between Hif1α and *foxO4* under hypoxic conditions. It was found that the expression of *foxO4* was up-regulated in ZF4 cells and zebrafish tissues after hypoxia treatment, and Hif1α could directly target the HRE of the *foxO4* promoter to regulate *foxO4* transcription, indicating that *foxO4* was involved in the hypoxia response by the Hif1α-mediated pathway. Furthermore, we obtained *foxO4* knockout zebrafish and found that the disruption of *foxO4* increased the tolerance to hypoxia. Further research found that the oxygen consumption and locomotor activity of *foxO4*^−/−^ zebrafish were lower than those of WT zebrafish, as was true for NADH content, NADH/NAD^+^ rate, and expression of mitochondrial respiratory chain complex-related genes. This suggests that disruption of *foxO4* reduced the oxygen demand threshold of the organism, which explained why the *foxO4^−/−^* zebrafish were more tolerant to hypoxia than WT zebrafish. These results will provide a theoretical basis for further study of the role of *foxO4* in the hypoxia response.

## 1. Introduction

Oxygen is an indispensable element in life that plays as a key role in cellular energy production and metabolism. The maintenance of normal growth and reproduction of oxygen-consuming organisms is inseparable from an environment with sufficient oxygen [1]. The mitochondrial electron transport chain (ETC) mediates a stepwise electron flow from NADH or succinate to molecular oxygen through a series of electron carriers (complex I–IV), and ADP is phosphorylated to form ATP in the process. Oxygen obtained through respiration is utilized as the terminal electron acceptor on ETC complex IV, which accepts electrons from cytochrome *c* and combines with protons (H^+^) to generate H_2_O [2].

However, hypoxia is a widespread phenomenon and can occur not only as a consequence of low atmospheric oxygen tension, but also at sites of inflammation, tissue injury, and ischemia, as well as in cancers [3]. Therefore, organisms have evolved multiple defense mechanisms that are activated under hypoxic conditions and the potentially acute or chronic adverse health consequences of hypoxia [3]. Among the complex hypoxia response mechanisms, the hypoxia response pathway involving hypoxia-inducible factor (HIF) is the most classical and the most thoroughly studied. Hypoxia leads to the activation and protein stabilization of HIF1α, which in turn activates many downstream genes of HIF1α involved in important biological processes, such as erythropoiesis, angiogenesis, and glycolysis. Some genes in involved in these processes include *EPO*, *VEGF*, *IGFR*, and *GLUT1* [4,5,6]. Currently, although many genes have been reported to be directly or indirectly involved in hypoxia signaling pathways, only a few of these genes have been investigated for their roles in hypoxia tolerance and hypoxia adaptation in aerobic organisms [7].

The FoxO signaling pathway is considered to be one of the main regulatory pathways in hypoxic stress [8,9]. In *Caenorhabditis elegans*, the ability of *daf-2* (*e1370*) larva and adult to survive high-temperature hypoxia or long-term hypoxia was demonstrated to depend on DAF-16 (Orthologs of all *foxO* family members) [10]. Similarly, FoxO3 has also been shown to modulate hypoxia signaling in humans and zebrafish via upregulating the von Hippel-Lindau tumor suppressor gene, and the destruction of the *foxO3* gene is not conducive to survival of zebrafish under hypoxic conditions [7]. For FoxO4, like other FoxO transcription factors, it structurally has the conserved FHD and TAD domains, and functionally regulates a number of genes involved in a variety of biological processes, including metabolism, cell proliferation, apoptosis, and immunity, among others. [11]. *FoxO4* is found to have only one duplicated copy, unlike *FoxO1*, *FoxO3*, and *FoxO6*, which each have one duplicated copy in mammals and two duplicated copies in catfish and zebrafish [12]. However, its roles in hypoxia adaptation in animals are unclear.

Therefore, in this study, we examined the expression of *foxO4* in a hypoxic environment and determined the regulatory relationship of Hif1α to *foxO4*. In addition, we constructed a *foxO4* zebrafish knockout line, in which we found that the disruption of *foxO4* reduced the oxygen demand of zebrafish and increased zebrafish hypoxia tolerance. These results could help accelerate the progress of research on the hypoxic adaption mechanisms of teleosts.

## 2. Results

### 2.1. Hypoxia Leads to Up-Regulation of foxO4 mRNA Expression

**Figure 1:**
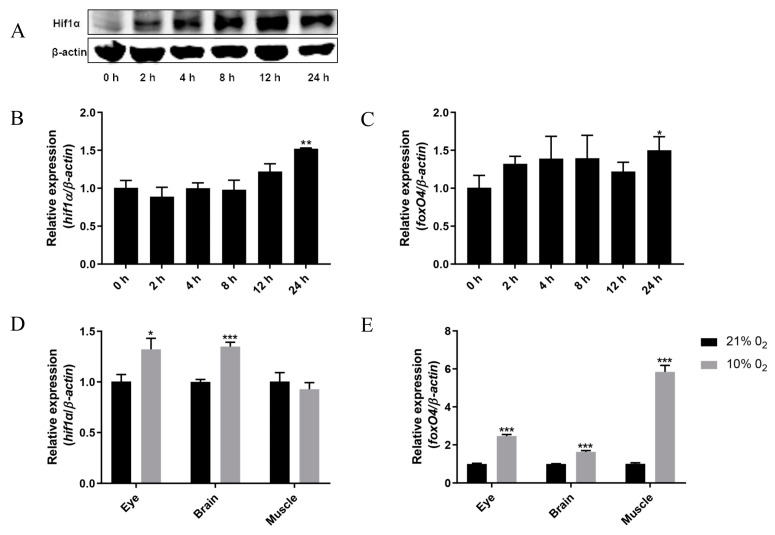
Effect of hypoxia on the expression of *foxO4* mRNA. (**A**) Protein detection of Hif1α in ZF4 cells treated with 100 μM CoCl_2_ for 0 to 24 h. (**B**,**C**) Expression of *hif1α* and *foxO4* in ZF4 cells treated with 100 μM CoCl_2_ for 0 to 24 h. (**D**,**E**) Expression of *hif1α* and *foxO4* in adult zebrafish treated with 10% O_2_ for 24 h.* *p* < 0.05, ** *p* < 0.01, *** *p* < 0.001.

In a CoCl_2_ hypoxia simulating experiment of ZF4 cells [13,14,15], we found that in addition to the up-regulation of Hif1α protein and mRNA levels (Figure 1A,B), *foxO4* mRNA levels were significantly increased after CoCl_2_ treatment for 24 h (Figure 1C). The same was true in vivo, where hypoxia resulted in up-regulation of *foxO4* mRNA levels in the eye, brain, and muscle of zebrafish (Figure 1E). This result indicate that hypoxia led to the up-regulated expression of *foxO4* mRNA.

### 2.2. Hif1α Directly Targets the foxO4 Promoter under Hypoxia

**Figure 2:**
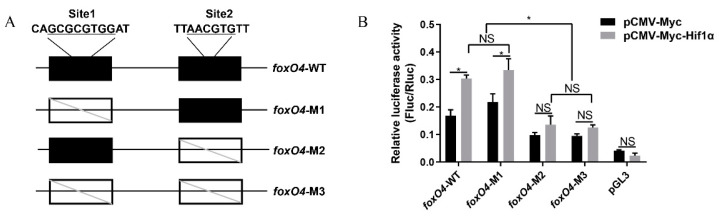
Luciferase reporter gene assay validates Hif1α targeting *foxO4*. (**A**) A schematic depiction of the *foxO4* promoter region containing two putative Hif1α binding sites. Underlined letters indicate hypoxia response element sequence. Black boxes indicate sites. Blank boxes indicate disrupted sites. (**B**) Luciferase reporter assay for *foxO4* promoter constructs. NS: Not significant. * *p* < 0.05.

The central element of the hypoxia response is the transcription factor Hif1α. To validate whether the marked upregulation of *foxO4* under hypoxic conditions is mediated through the canonical Hif1α signaling pathway, hypoxia response elements within the 2 kb region upstream of *foxO4* were analyzed with the JASPAR database. As shown in Figure 2A, two hypoxia response elements were predicted, site 1 (GCGCGTGG) and site 2 (AACGTG), and the luciferase reporter gene assay in HeLa cells showed that the overexpression of zebrafish Hif1α protein significantly promoted the transcription activity of the *foxO4* promoter, but this promotion disappeared when site 2 was mutated (Figure 2B). The results indicate that *foxO4* was directly regulated by Hif1α to participate in the hypoxia response.

### 2.3. Disruption of Zebrafish foxO4 Increases Hypoxia Tolerance

**Figure 3:**
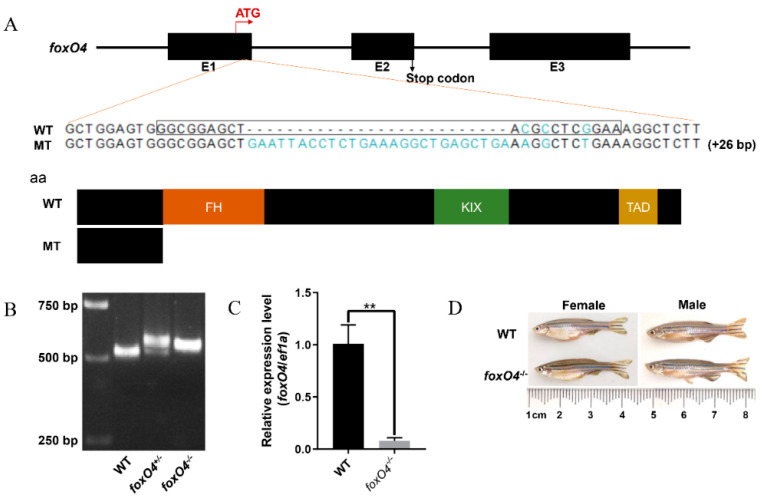
Generation of *foxO4*^−/−^ zebrafish via CRISPR/Cas9 technology. (**A**) The DNA sequence and predicated protein product difference in *foxO4* between the mutants and WT. sgRNA sequence is marked with a black box. The green letters in MT represent the mutated DNA sequence. (**B**) Verification of zebrafish *foxO4* disruption by agarose gel electrophoresis. (**C**) *foxO4* mRNA expression levels in *foxO4*^−/−^ and WT zebrafish embryos at 96 hpf were detected by qPCR. ** *p* < 0.01. (**D**) Observation of the *foxO4*^−/−^ and WT adult zebrafish.

To facilitate the study of *foxO4* function in the hypoxia response, we used CRISPR/Cas9 technology to construct the *foxO4* mutant. After screening, a *foxO4* gene mutant with an insert of 26 bp was obtained, in which the translation of the FoxO4 protein was prematurely terminated (Figure 3A). qPCR analysis revealed that the expression of *foxO4* mRNA was significantly reduced in the mutant compared with the WT (Figure 3C), indicating that *foxO4* had been successfully knocked out in the mutant. *foxO4*^−/−^ zebrafish were observed to develop normally and were generally indistinguishable from the WT zebrafish (Figure 3D).

**Figure 4:**
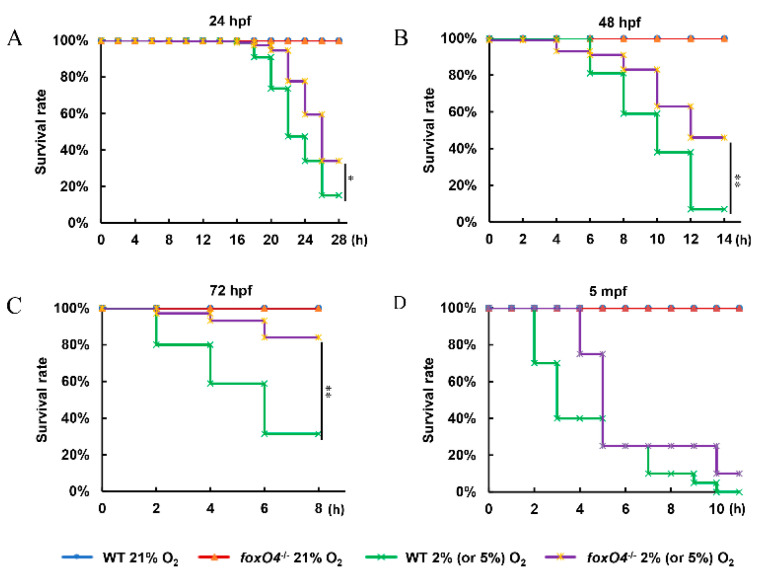
Survival rate curves. Hypoxia treatment with 2% O_2_ was performed in *foxO4*^−*/*−^ and WT zebrafish at different development stages with 24 hpf (**A**), 48 hpf (**B**) and 72 hpf (**C**). Hypoxia stress in 5 mpf adult zebrafish (**D**) with 5% O_2_. hpf: day after fertilization. mpf: month after fertilization. * *p* < 0.05, ** *p* < 0.01, * or ** represents the difference in the overall survival curve.

The biological consequences of *foxO4* participating in hypoxia signaling were investigated by comparing acute hypoxia tolerance in the *foxO4*^−/−^ and WT zebrafish. *foxO4*^−/−^ embryos (24, 48, 72 hpf) had a higher survival rate than WT treated with 2% O_2_ at the same time points (Figure 4A–C). Similarly, for adult zebrafish (5 months old), more than half of WT showed a loss of equilibrium within 4 h of 5% O_2_, whereas *foxO4*^−/−^ zebrafish did not show a loss of equilibrium until 4 h later (Figure 4D). These data suggest that deletion of *foxO4* favors zebrafish survival under hypoxic conditions.

### 2.4. Disruption of Zebrafish foxO4 Reduces Oxygen Demand

**Figure 5:**
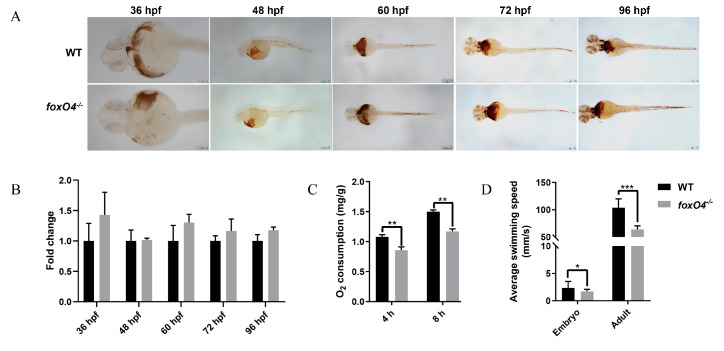
Hemoglobin, oxygen consumption and locomotor activity. (**A**) *O*-dianisidine staining for hemoglobin in *foxO4*^−*/*−^ and WT zebrafish embryos from 36 hpf to 96 hpf. Scale bar = 250 µm. (**B**) The ratio of total strength of *foxO4^−/−^* zebrafish staining signal to WT. ImageJ software was used to quantify the total strength of the staining signal of each embryo. (**C**) Oxygen consumption of *foxO4^−/−^* and WT adult zebrafish. (**D**) Locomotor activity of *foxO4*^−*/*−^ and WT zebrafish embryos and adults. * *p* < 0.05, ** *p* < 0.01, *** *p* < 0.001.

**Figure 6:**
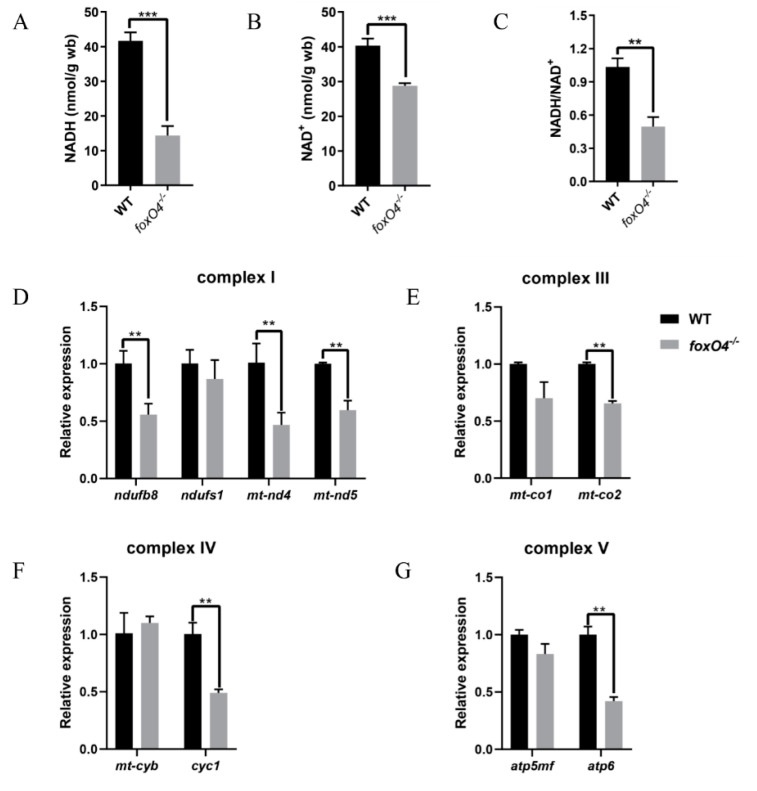
NAD(H) content and expression of mitochondrial respiratory function-regulated genes. NADH (**A**) and NAD^+^ (**B**) contents and NADH/NAD^+^ rate (**C**) in muscle tissue of *foxO4^−/−^* and WT adult zebrafish. Expression of genes associated with mitochondrial respiratory complexes I (**D**), III (**E**), IV (**F**) and V (**G**). Gene expression level was normalized to *ef1a*. ** *p* < 0.01, *** *p* < 0.001.

To explore the reason why the *foxO4*^−*/*−^ zebrafish are more tolerant to hypoxic conditions, the oxygen consumption of adult zebrafish was measured. It was found that the oxygen consumption of the WT was significantly higher than that of the *foxO4*^−/−^ zebrafish (Figure 5C), while no abnormality was found in hemoglobin content of *foxO4*^−/−^ zebrafish (Figure 5A,B). In addition, in the absence of external stimulation, the swimming speed of *foxO4*^−/−^ zebrafish was slower than that of WT (Figure 5D), and *foxO4*^−/−^ zebrafish had lower NADH and NAD^+^ contents and NADH/NAD^+^ rate than WT (Figure 6A–C). The same was true for genes regulating mitochondrial respiratory function, which had an overall low expression level in *foxO4*^−/−^ zebrafish (Figure 6D–G). These results suggest that the *foxO4*^−/−^ zebrafish has a relatively low oxygen demand, and thus the oxygen concentration threshold required for survival is lower than that of WT zebrafish.

## 3. Discussion

In recent years, the FoxO family has been recognized as an important transcriptional regulator involved in a variety of cellular functions, among which is the response to hypoxia [16]. Due to redundant functions among members of the FoxO family, functional studies of *FoxO4* are easily overlooked, especially in the regulation of hypoxia in organisms. Therefore, in this study, the model organism zebrafish was used as the research object to explore the role of *foxO4* in the hypoxia response.

Research on *FoxO4* in hypoxia has mainly focused on the hypoxic microenvironment of cancer cells. In cancer cells, FoxO4 is more prone to low expression, and its expression is negatively correlated with the presence of lymph node metastasis and tumor diameter [17,18]. There are also reports of FOXO4 downregulation in cancer cells treated with 1% O_2_ or CoCl_2_ [18]. However, in mouse 3T3L1 cells, hypoxia leads to increased transcription of the *FoxO4* gene and increased nuclear activity of FoxO4 [19]. In this study as well, the expression of *foxO4* was altered and significantly up-regulated after ZF4 cells were treated with CoCl_2_ or zebrafish were treated with 10% O_2_.

Hif1α is an important regulator of oxygen homeostasis. It was found in our experiments that the response of *foxO4* to hypoxia followed the massive accumulation of Hif1α protein in ZF4 cells. Whether *foxO4* is regulated by Hif1α and participates in the Hif1α signaling pathway is unclear. Therefore, we verified using a luciferase reporter gene assay and found that Hif1α increases the transcriptional activity of zebrafish *foxO4* under hypoxia, demonstrating that Hif1α can directly target *foxO4*. Additionally, FoxO4 also plays a role in inducing downregulation of HIF1α via a VHL protein-independent mechanism, and it has been shown to directly negatively regulate HIF1α in gastric cancer cells, thereby inhibiting various responses to hypoxia [4,18]. These indicate that the hypoxia regulation mechanism is very complex, and, under hypoxic conditions, *FoxO4* can be up-regulated by Hif1α, and can also regulate Hif1α by negative feedback.

In mice, conditional deletion of *FoxO1*, *FoxO3*, and *FoxO4* was found to affect formation of hematopoietic stem cells and neural stem/progenitor cells [20,21], and compromised pancreatic *β* cell function, leading to maturity-onset diabetes of the young (MODY)-like diabetes [22]. Studies also show that these three genes play a role in atherosclerotic lesion formation [23,24]. However, mice lacking only *FoxO4* were completely indistinguishable from their littermate controls, and histological analysis did not reveal any consistent abnormalities [25]. In zebrafish as well, disruption of *foxO4* did not affect normal development. However, when treated with 2% or 5% O_2_, it was found that both *foxO4*^−/−^ zebrafish embryos and adults exhibited more hypoxia tolerance than WT.

It is not surprising that overexpression of FoxO4 also has adverse consequences, although activated *FoxO4* appears to play a role in maintaining homeostasis during hypoxia. FOXO4 is thought to provide a new pathway for carcinogenesis, as hypoxia-induced FOXO4 expression may contribute to the induction of unfavorable factors in tumorigenesis, such as PAI-1 [16]. Furthermore, the overexpression of FoxO4 also regulates key factors in the apoptosis pathway, such as Bcl-2 and Bcl6, among others, aggravating cell apoptosis [26]. FoxO4 can also negatively regulate USP10 transcription by blocking the Hippo/YAP pathway, aggravating H/R-induced apoptosis and oxidative stress in H9C2 cells [27]. Likewise, knockdown of FoxO4 significantly protected rats from myocardial I/R injury [28,29].

Of course, we explain the hypoxia-tolerant phenotype of *foxO4^−^*^/*−*^ zebrafish from another perspective; that is, the oxygen consumption of the organism. As an important carrier of oxygen transport through blood in vertebrates, erythrocytes play an important role in cellular respiration and cellular metabolism of aerobic organisms. In this research, it was found that the oxygen consumption of *foxO4^−^*^/*−*^ zebrafish was significantly lower than that of WT, but this did not appear to be related to the oxygen transport carrier, because the erythrocyte content in the *foxO4^−^*^/*−*^ zebrafish was not abnormal.

Furthermore, reduced locomotor activity was found in both *foxO4*^−/−^ zebrafish embryos and adults under normal conditions, implying lower levels of body metabolism. This is reminiscent of turtles, whose low metabolic rates have been endowed by long-term natural selection, allowing them to survive in extremely harsh hypoxic environments [30]. A reduction in the NADH/NAD^+^ ratio usually represents a decrease in mitochondrial function and cellular metabolism [31]. It can be speculated that the destruction of *foxO4* reduces the metabolic level of the body and reduces the oxygen demand, which was also confirmed by the reduced NADH and NAD^+^ contents, as well as the decreased NADH/NAD^+^ ratio and expression level of mitochondrial respiratory complex-related genes in *foxO4*^−/−^ zebrafish. Thus, *foxO4*^−/−^ zebrafish with a low oxygen demand threshold are more tolerant to hypoxic conditions than WT zebrafish. As for the mechanism of how the destruction of *foxO4* leads to the decline of the body’s metabolic level, further research is needed in the future.

## 4. Materials and Methods

### 4.1. Cell Culture

The zebrafish embryonic fibroblast cell line ZF4 was cultured in DMEM/F12 (1:1) medium (Gibco, Waltham, MA, USA), supplemented with 10% fetal bovine serum (FBS) at 28 °C. The HeLa cell line was grown in DMEM medium (Gibco) and 10% FBS at 37 °C.

### 4.2. Hypoxia Treatment

Hypoxia treatment was performed in a Ruskinn Invivo2 300 work station. The culture water was put into the chamber for hypoxia treatment 6 h before the experiment. In the hypoxia chamber, each period of 300 zebrafish embryos of each type was divided equally into six 90 mm petri dishes with 15 mL of treated culture water. Each group of 20 adult zebrafish (0.35 ± 0.03 g) was divided equally into four 500 mL flasks with 500 mL of treated culture water. Zebrafish embryos and adults were treated in chambers with oxygen concentrations of 2% and 5%, respectively, and a water temperature of 28 °C, and then the dead embryos or adults with loss of equilibrium were counted every 2 h. The experiment was repeated three times. This study was conducted in accordance with ethical standards and according to the national and international guidelines, and approved by the Scientific Ethic Committee of Huazhong Agricultural University (Wuhan, China) (HZAUFI-2020-0022).

To simulate hypoxic condition, ZF4 cells cultured in 6-well plates were treated with CoCl_2_ for 0 (control), 2, 4, 8, 12, and 24 h before being collected for isolation of total RNA and protein, as described by Huang et al. [32].

### 4.3. Plasmid Constructs

The hypoxia response element on the 2 kb region upstream of *foxO4* was predicted using the JASPAR CORE Vertebrate database. To verify the transcription activity, wild type *foxO4* promoter fragments containing predicted hypoxia response elements and mutants of hypoxia response elements (Figure 2A) by site-directed mutagenesis were inserted into the luciferase reporter vector pGL3-Basic (Promega, Madison, WI, USA), respectively. The construction of the pCMV-Myc-Hif1α expression vector has been described previously [32]. The primers used are listed in Table S1.

### 4.4. Cell Transfection and Luciferase Reporter Assays

For verification of hypoxia response element activity, HeLa cells were seeded in 24-well plates and transfected with the indicated plasmids using Lipofectamine 2000 (Invitrogen, Waltham, MA, USA) according to the manufacturer’s protocol. The cells were then treated with CoCl_2_ (100 µM) for 4 h before the luciferase assay was conducted [32]. After 24 h of transfection, firefly and *Renilla* luciferase activities were measured using the Dual-Luciferase Reporter Assay System (Promega).

### 4.5. Generation of foxO4 Mutant Zebrafish

Disruption of *foxO4* in zebrafish was accomplished using CRISPR/Cas9 technology. The sgRNA target site of zebrafish *foxO4* was designed by the CRISPR/Cas9 target online predictor (CCTop, http://crispr.cos.uni-heidelberg.de (accessed on 12 November 2019)). The template of sgRNA was amplified by specific primers, as shown in Table S1, and the sgRNA was synthesized using a TranscriptAid T7 Hight Yield Transcription kit (Thermo Fisher Scientific, Waltham, MA, USA) according to the manufacturer’s instructions. The knockout experiment in zebrafish embryos was performed as described previously [13,33]. For mutant detection, the genomic region flanking the sgRNA target site was amplified, followed by sequencing of the PCR products to identify the genotype. The positive F0 zebrafish were backcrossed with the wild-type (WT) zebrafish to generate F1. Then, homozygotes (−/−) were screened from F2 offspring produced by crosses of F1 adult zebrafish with the same genotype (+/−).

### 4.6. Oxygen Consumption and Locomotor Activity

For the oxygen consumption experiment, *foxo4^−/−^* and WT zebrafish (0.35 ± 0.03 g, *n* = 5 each glass flask) were individually put into six 500 mL glass flasks with 500 mL of culture water (the water from the zebrafish circulatory system was autoclaved and fully aerated). Following the protocol [34], the oxygen concentration of water in the flasks was measured with a dissolved oxygen meter (INESA, Shanghai, China) at three time points before putting in zebrafish 4 h and 8 h after sealing. Oxygen consumption was calculated as accumulated oxygen uptake of zebrafish divided by body weight.

*foxO4^−/−^* and WT zebrafish embryos (125 hpf, *n* = 24 each) were individually transferred into single wells of 24-well polystyrene plates with 1620 µL culture water and were then analyzed using a DanioVision system (Noldus, Wageningen, The Netherlands) monitoring enclosure with corresponding software in tracking mode (EthoVision XT14, Noldus, Wageningen, The Netherlands) for 15 min. Each adult zebrafish (0.35 ± 0.03 g) was tracked for 15 min using a camera in the water tank (30 × 30 cm) and analyzed with EthoVision XT14 software. To evaluate the locomotor activity, the average speed was compared in the last 5 min.

### 4.7. O-Dianisidine Staining

Zebrafish embryos at different developmental stages were collected and fixed with 4% paraformaldehyde. Their hemoglobin was stained with *O*-dianisidine (Sigma-Aldrich, Shanghai, China) following a previously established protocol [35].

### 4.8. NAD(H) Content

Muscle of adult zebrafish was assayed for NAD(H) content according to the manufacturer’s instructions (Solarbio, Beijing, China).

### 4.9. Quantitative Real-Time PCR (qRT-PCR)

Total RNA in cells and eye, brain, and muscle of adult zebrafish was extracted using Trizol and reverse transcribed using HiScript^®^ II reverse transcriptase (Vazyme, Nanjing, China). The target gene expression was determined by quantitative real-time PCR (qPCR) assays. qPCR was performed in a 20 µL reaction mix containing 7.4 µL of nuclease-free water, 1 µL of cDNA template, 0.8 µL of each primer (10 µM), and 10 µL of monamp^TM^ SYBR^®^ green mix (Monad, Wuhan, China). Three replicates were set. The qPCR program consisted of an initial denaturation at 95 °C for 30 s, followed by 40 cycles of 95 °C for 10 s, 60 °C for 10 s, and 72 °C for 20 s. The gene expression level was calculated using the 2^−∆∆Ct^ method. All primers are listed in Table S1.

### 4.10. Western Blotting

ZF4 cells were lysed in RIPA lysis buffer (Beyotime Biotechnology, Shanghai, China) with added PMSF (Beyotime Biotechnology, Shanghai, China). The protein concentration was determined using the BCA protein assay kit (Biosharp, Beijing, China). Protein samples were analyzed by Western blotting as described previously [36]. Primary antibodies used were anti-Hif1α (1:500, polyclonal antibody against zebrafish Hif1α made in our laboratory) and anti-ACTB (1:100,000, ABclonal, Wuhan, China).

### 4.11. Statistical Analyses

All data were presented as mean ± SD. Statistical analyses were performed using *t*-tests or one-way ANOVA followed by Duncan’s tests, under which *p* < 0.05 was considered significant.

## 5. Conclusions

In summary, zebrafish *foxO4* is involved in hypoxic stress by a Hif1α mediated pathway. Furthermore, the *foxO4* knockout zebrafish mutant was obtained and showed developmentally indistinguishable changes compared with WT zebrafish. However, disruption of *foxO4* reduced the oxygen demand of zebrafish, which explains the conclusion that *foxO4^−/−^* zebrafish were more tolerant to hypoxia than WT zebrafish.

## Data Availability

All datasets generated for this study are included in the article and the Supplementary Materials.

## Supplementary Materials

The supporting information can be downloaded at: https://www.mdpi.com/article/10.3390/ijms24108942/s1.
