# Supplementary material for: Deletion of the foxO4 Gene Increases Hypoxia Tolerance in Zebrafish
Source: Int J Mol Sci. 2023 May 18;24(10):8942. doi: 10.3390/ijms24108942 (PMC10218838; doi:10.3390/ijms24108942)
Supplement: Supplementary file 1 [file ijms-24-08942-s001.zip › ijms-2297504-supplementary.pdf]

International Journal of
Molecular Sciences

MDPI

Table S1. Primers used in this experiment.

Primer name	Sequence (5'-3')	Use of Primers
foxO4-sgRNA-F	GTAATACGACTCACTATAGGCGGAGCTACGCCTCGGAAGTTTTAGAGCTAGAAATAGC	Amplified sgRNA template
foxO4-sgRNA-R	RAAAAGCACCGACTCGGTGCCACTTTTTCAAGTTGATAACGGACTAGCCTTATTTTAACTTGCTATTTCTAGCTCTAAAAC	
foxO4-sg-F	CGCCTCAAAACTGATTCACTTA	Mutant detection
foxO4-sg-R	GAACTGTTACTATCTCCTTTGTCC	
foxO4-WT-F1	ctagctagcACTGTATCCTCTACCGCCCAC	Plasmid construction
foxO4-WT-R1	tcccccgggGTGAATCAGTTTTGAGGCGATA	
foxO4-MT-R1	CACTACTGCAATAATAACATATATGGAGTGACGG	
foxO4-WT-F2	TATTGCAGTAGTGGCTCTTGAC	
foxO4-MT-R2	tcccccgggGTGAATCAGTTTTGAGGCGATATTAAAATATCAACGCCAACAGACTGGGAGACAATTTAACAAAACATGGAAATAATCC	
foxO4-qF	GCCAGTACACTGAGCGGT	qPCR
foxO4-qR	GAGCCCATCAATCAAATCC	
hif1a-qF	ACTGTTTGCTATTGATACCG	
hif1a-qR	AGGAGGGTAAGGGTTGG	
ndufb8-qF	GGTTCTTCTAAGGACAGTCT	
ndufb8-qR	CAGCCATCATCAGGATAGG	
ndufs1-qF	GCGAGCAATATGGTTGAAG	
ndufs1-qR	AGACACATACGGCAGTTAC	
mt-nd4-qF	CAGGGTGAAATGCTTCTAAC	
mt-nd4-qR	TTACAGGCTCCGAGTTGA	
mt-nd5-qF	ATCCCACTTACAATGATGCT	
mt-nd5-qR	AGGTGATGAATAAGGCGATT	
mt-co1-qF	GACCGCATTAAGCCTCTT	
mt-co1-qR	TTAGTGGCACAAGTCAGTT	
mt-co2-qF	CGCAGCATCACCTGTAAT	
mt-co2-qR	GGAAGGACTGTTCATACGAT	
mt-cyb-qF	CAATACACTACACCTCAGACA	
mt-cyb-qR	ACAGGCAGATGAAGAAGAAG	
cyc1-qF	TGTTGACGACAGGAGGAG	
cyc1-qR	AGGACAGAAAGCCACCAT	
atp5mf-qF	TCGGAACAAGAGACTTCAC	
atp5mf-qR	CAACAAGGAACATGGCAAT	
atp6-qF	TTATCCTCGTTGCCATACTT	
atp6-qR	TTGTGAATCGTCCAGTCAA	
β-actin-qF	AGAGCTATGAGCTGCCTGACG	
β-actin-qR	CCGCAAGATTCCATACCCA	
ef1a-qF	CTTCTCAGGCTGACTGTGC	
ef1a-qR	CCGCTAGCATTACCCTCC	
